# Remini-Sing: A Feasibility Study of Therapeutic Group Singing to Support Relationship Quality and Wellbeing for Community-Dwelling People Living With Dementia and Their Family Caregivers

**DOI:** 10.3389/fmed.2018.00245

**Published:** 2018-08-31

**Authors:** Jeanette Tamplin, Imogen N. Clark, Young-Eun C. Lee, Felicity A. Baker

**Affiliations:** Faculty of Fine Arts and Music, University of Melbourne, Melbourne, VIC, Australia

**Keywords:** group singing, music therapy, dementia, family caregivers, community, feasibility, quantitative assessment

## Abstract

**Background:** Living at home following a diagnosis of dementia can be difficult for both the person living with dementia (PwD) and their family caregivers (FCG). Active group music participation may provide an avenue for emotional release, offer psychosocial support to caregivers and stimulate meaningful interaction between caregivers and loved ones with dementia. Therapeutic music interventions also have the capacity to facilitate reminiscence and social engagement and can help to manage challenging symptoms associated with dementia, such as anxiety, apathy, and agitation.

**Method:** This feasibility study examined the acceptability of a 20-week therapeutic group singing intervention (Remini-Sing) and quantitative research assessments for PwD/FCG dyads living in the community. Quantitative measures for the following outcomes were tested for sensitivity and acceptability: relationship quality (PwD and FCG); life satisfaction, caregiver satisfaction, flourishing, and depression for FCGs; and anxiety, apathy, agitation, and quality of life for PwD. Quantitative assessments were conducted before, during (midway) and after 20 weeks of participation in a therapeutic singing group attended by the PwD and FCG together. The Remini-Sing intervention incorporated vocal warm ups, singing familiar songs, learning new songs, and opportunities for social interaction. Qualitative interviews were conducted with all dyads that completed the intervention.

**Results:** Twelve PWD/FCG dyads were recruited and enrolled in the study. High participation and retention rates indicated that the intervention was received favorably by participants. There were no statistically significant changes on measures from pre to post intervention. However, favorable baseline scores on relationship quality and wellbeing measures were sustained over the 20-week intervention. The testing of these measures for feasibility also revealed that some were too difficult for PwD and thus yielded questionable results, some were potentially less relevant, and there were likely floor and ceiling effects on several of the measures utilized.

**Conclusions:** This study demonstrated good feasibility for a research protocol and therapeutic group singing intervention for community-dwelling PwD and their FCGs. Participant reflections and researcher observations yielded useful information guiding the selection of quantitative outcome measures for future research in this area.

## Introduction

Dementia is a neurodegenerative condition that significantly compromises health and wellbeing for increasingly larger numbers of people as the global population ages. Current estimates indicate that around 47 million people worldwide are currently living with dementia, and this figure is projected to increase to 131 million by 2050 (1, 2). The effects of dementia are devastating for those living with the condition as well as their families and have significant economic consequences for entire health systems. A vast majority of people living with dementia (PwD) remain in the family home with support from informal co-resident primary caregivers, usually a spouse/partner or child (3–5). The societal global costs attributed to informal care provided by family caregivers (FCG) is estimated to be around US$330.8 billion (6). Remaining in the family home, with familiar people, objects, and memories, provides optimal environmental and care conditions for PwD and also reduces costs to society associated with residential care and this is thus advocated by the World Health Organization (7).

Relationship quality between the PwD and their FCG is recognized as a major factor that influences the health and wellbeing of both, and consequently impacts their ability to live together in the family home. For FCGs, the level of burden associated with caregiving can lead to negative physical and mental health including depression, fatigue, burnout, and illness (8) with FCGs of community-dwelling PwD exhibiting higher rates of mental illness and lower subjective welling than the general population (9). Symptoms associated with dementia such as language and memory impairments, together with the stigma of dementia can lead to avoidance of previously meaningful activities and social withdrawal (10, 11). Social isolation is also common for FCGs due to the responsibilities of full time care and their loved one's unpredictable behavior during social situations (7, 12). Programs that aim to support emotional coping for FCGs, independence for PwD, and health, wellbeing and social participation for both, can delay admission to residential care for PwD, especially if commenced early in the disease progression (7, 13, 14). In particular, there is a demonstrated need for supportive, strengths-based interventions that PwD and their FCG can participate in together; interventions that focus on supporting their relationship and social connectedness rather than just their individual needs (12, 15, 16).

Music therapy offers an opportunity for strengths-based, dyad friendly intervention (17, 18). Areas of the brain responsible for processing music are retained until late in the trajectory of dementia (19, 20). Therefore, active music interventions offer benefits that can support PwD/FCG dyads living in the community. Specifically:

1. Memories for music and song lyrics are relatively preserved in PwD into the late stages of dementia, which can increase engagement and stimulate successful social interaction (19, 20)
2. Music preferences and associations are individual for each person and therefore reveal the premorbid personality of PwD to the FCG (21)
3. Music interventions can assist in the management of negative symptoms of dementia including depression, agitation, anxiety, and apathy (22, 23).

These positive outcomes offer opportunities for interpersonal connection between PwD and their FCGs, coping strategies for FCGs, and experiences of empowerment and independence for PwD. It is possible that other social activities (such as cooking, craft, or dancing) might also function as a focus for dementia support groups. However, the almost universal appeal of music (with its social, emotional rewards), together with the accessibility of singing throughout the dementia trajectory, make it an ideal focus for group intervention in dementia.

Although the media is awash with observational and anecdotal accounts of the benefits of music participation for PwD, there are few rigorous studies that support these claims.

Recent randomized controlled trials have reported improvements in cognitive function, mood (24) and depression (25) following music interventions for PwD. A recent Cochrane review of music therapy for PwD found 10 studies reporting positive effects, however these studies were limited by small sample sizes, short intervention periods, poor methodological quality, and heterogeneous outcomes (23). Previous research examining the effects of music therapy interventions in dementia care has often focused on PwD living in residential care facilities and has not considered FCGs. Studies that have examined the effectiveness of music interventions for PwD living in the community have suggested improvements in quality of life but exhibited similar methodological issues to those cited above (26–28).

The aim of this study was to determine the feasibility of delivering and measuring the effects of a therapeutic group singing and home-based music program on the primary outcome of relationship quality and secondary wellbeing outcomes for PwD and their FCGs. We wanted to test the feasibility of the study protocol, establish the appropriateness of the measures for answering the research questions, and collect pilot data to determine sample size for a randomized controlled trial. We also wanted to collect qualitative data through interviewing participants to gather information about their experience of the choir, home music program, and quantitative research measures. The study was co-funded by the Australian National Health and Medical Research Council and the Australian Research Council (APP1106603). Ethics clearances were provided by the Austin Health Human Research Ethics Committee (HREC/15/Austin/445) and written informed consent was obtained from all participants. The study was also registered with the Australian New Zealand Clinical Trials Registry (ACTRN12618001059257).

## Materials and methods

### Study design

This feasibility study used a prospective, pre-post design to test the uptake and accessibility of a 20-week therapeutic group singing protocol and home-based music program. The study was also designed to test the sensitivity, acceptability, and appropriateness of quantitative wellbeing measures for the PwD and FCG. We incorporated interviews with participants to better understand their experience of both the music therapy intervention and the assessment process in order to refine these for future research.

### Participants

People living with dementia in the community (and their FCGs) were referred to the study by the Austin Health Cognitive Dementia and Memory Service and Aged Care Assessment Services, and also by community dementia services in Melbourne, Australia. We provided information sessions to groups targeting potential participants and distributed an information flyer to community dementia groups. Interested potential participants were screened against the following inclusion criteria. PwD/FCG dyads had to be living in their own home in the community to be eligible for this study. PwD were eligible for the study if they had a clinical diagnosis of dementia of any type (based on geriatric/neuropsychological assessment) and a Mini Mental State Exam score between 10 and 26 (29). FCG could be a spouse/partner, child or other family member and were eligible for the study if they were the primary caregiver for the PwD, and. Both PwD and FCG participants needed to have adequate or assisted hearing and sufficient English to complete the research measures. Participants also needed to be available to attend the weekly Remini-Sing sessions at the scheduled time. No prior musical experience was required for either PWD or FCG participants.

### Remini-sing music therapy intervention and rationale

Remini-Sing is a once weekly 2-h therapeutic group singing program co-facilitated by two trained music therapists (authors 1 and 2). In this pilot feasibility study, we examined outcomes over a 20-week intervention period. Sessions were held in a spacious room at a large public health facility in Melbourne, Australia. The Remini-Sing intervention was modeled on prior research by our team (30) as well as previous music therapy studies in residential dementia care reporting improvements in confidence, motivation, depression, communication and social interaction following group singing-based music therapy interventions (25, 31–34). Based on our previous research (30), we aimed for a minim of 3 dyads and maximum of 15 dyads to facilitate the group process. Our Remini-Sing model is based on therapeutic group singing as a form of community music therapy for people living with dementia and their family caregivers. Kitwood's 12 concepts of person-centered care (35) were used to guide the facilitation of Remini-Sing sessions [as described elsewhere (36)]. As such, the Remini-Sing model is person-centered, strengths-based and process-oriented, rather than product or performance oriented, which distinguishes it from dementia-friendly community choir models (26–28). Remini-Sing is designed to utilize the normalizing, stimulating, and accessible properties of group singing to improve or maintain personal relationships, social engagement, and emotional wellbeing.

The music therapist facilitators provide a therapeutic program utilizing variety of singing-based activities targeted to enhance memory, communication, wellbeing, and group cohesion. The session structure consisted of introductions and information updates (5–10 min), vocal warm ups and exercises (15–20 min), singing familiar, participant-requested songs (30–45 min), learning new songs, harmony parts, rounds, and singing skills introduced by the researchers (20–30 min), and social interaction and peer support over afternoon tea (30 min). The warmups consisted of breathing exercises, vocalizes with melodic and rhythmic variation, and physical stretching and balance exercises. These were conducted by one music therapist with supporting music provided via keyboard by the other music therapist. Songs were accompanied with live music on keyboard, guitar, and or banjo by one or both of the music therapists. Some songs and rounds were sung acapella. Familiar songs were suggested by participants during the sessions and new songs and rounds were also introduced by the music therapists. Song keys were adjusted during sessions as needed to match the comfortable vocal range of the group. Songs were generally only sung once per session, but frequently repeated from week to week depending on participant request and when performances were imminent. Simple variation of musical elements was introduced as the groups became more comfortable with singing together (e.g., dynamic variation, simple harmonies, movement to music). Some of the practical elements of the Remini-Sing model include: provision of name tags (to aid memory and social interaction), appropriate seating and positioning (chairs with arms if needed, and chairs arranged so that participants can see one another), adequate space for mobility aids and accessible toilet facilities, and large screen projection of lyrics rather than printed booklets (to avoid poor posture and confusion in finding song pages, and also to encourage eye contact and social engagement).

We also aimed to provide resources for a home music program designed to help FCGs manage the behavioral and psychological symptoms of dementia with their loved one. We conducted a music assessment with dyads at baseline to identify personally salient music for the construction of three playlists (this information was also used to identify song preferences for use in Remini-Sing sessions). The playlists were designed to facilitate: (1) Reminiscence, (2) Relaxation, and (3) Agitation reduction/calming down. We worked with participants to generate personalized 10 song playlists and provided these on CD to participants as this was their most commonly utilized method to access music. Music on the reminiscence playlist included songs that had marked important moments in the participants' lives, e.g., songs from special occasions such as weddings, or songs that reminded them of important people (spouse, family members, friends) and significant times/events (living overseas, traveling, family holidays). The relaxation playlist incorporated music that participants personally found relaxing and included new and familiar songs. The “calming down” playlist utilized the Iso principle (37) and thus began with high-energy familiar music to match feelings of irritability or agitation. Successive pieces of music on the playlist became gradually less stimulating to acknowledge and mirror current feeling states as well as encourage reduction in agitation. We provided education to FCGs on how they could use music to manage dementia symptoms at home with their loved one.

### Outcome measures

The feasibility of outcome measures was assessed by collecting data on the primary and secondary outcomes at baseline, midway through the intervention period (11 weeks), and post intervention (21 weeks). All participants completed self-report measures for the primary outcome of relationship quality and the secondary wellbeing outcomes: apathy and quality of life (PwD), and carer satisfaction, life satisfaction, depression and flourishing (FCG). The FCGs also completed caregiver ratings of PwD apathy, agitation, and quality of life. If the PwD was unable to complete the self-report measures then this data was not collected.

All assessments were conducted by a research assistant with the necessary training to complete these measures. Assessments took place either directly before or after a scheduled group session, or at a separate convenient time at the participant's home. In order to establish baseline demographics we completed the Dementia Rating Scale-2 (DRS-2) (38) at baseline only. The DRS-2 consists of 24 brief subtests whose scores are combined into five subscales of attention, initiation/perseveration, construction, conceptualization, and memory as well as an overall total score. All participants also completed the Mini Mental State Exam (MMSE) (29) to confirm eligibility for participation in the study. The MMSE is routinely used in dementia screening and assessment and has a maximum score of 30 points. A score of 20 to 24 suggests mild dementia, 13 to 20 suggests moderate dementia, and less than 12 indicates severe dementia.

#### Primary outcome—relationship quality

The primary outcome for this study was relationship quality, which was measured using the 14-item Quality of Carer Patient Relationship (QCPR) scale (39). The QCPR has demonstrated acceptable internal consistency (α = 0.82) and concurrent validity and has been used in intervention trials with PwD/CG dyads (39–41). The 14 items are scored on a 5-point rating scale with a total possible score range of 14–70. The QCPR is a self-report measure that was completed by both PwD and FCG about their perceived relationship quality.

#### Secondary wellbeing outcomes for the person living with dementia

*Anxiety* was measured using the Rating Anxiety in Dementia Scale (RAID) (42), a tool developed specifically for use with people with dementia. It includes 18 items designed to measure anxiety, each scored on a 4-point scale, with a total score range of 0–54. A score of 11 or more indicates significant clinical anxiety. Several items inquire about worry (worry about physical health, finances, etc.), while others include sleep disturbance, irritability, and a number of somatic symptoms (palpitations, dry mouth, shortness of breath). Information is gathered from a range of sources (PwD, FCG, and clinical observations) and collated by the clinician into a single score for each item. The RAID has fair to excellent interrater and test-retest reliability and satisfactory internal consistency (43).

*Apathy* was measured using the Apathy Evaluation Scale (AES), a 19-item measure designed to measure apathy in adult patients (44). Each item is scored on a 4-point scale, with a total score range of 19–76. The AES has demonstrated good reliability and validity and was developed for multiple rater sources. We used the self-rated and informant (FCG) versions. As apathy is conceptualized as a pathological construct, higher AES score indicate more apathy. Scores above 42 generally indicate minimal or mild apathy.

*Agitation* was measured using the caregiver-completed 14-item Cohen-Mansfield Agitation Inventory—Short Form (CMAI-SF) (45), which has good inter-rater reliability. The CMAI-SF does not provide a total score for agitation but rather gives frequency ratings for 14 agitated behaviors using 5-point scales.

The Quality of Life—Alzheimer's Disease (QoL-AD) tool (46) was used to measure *quality of life* for the PwD. The QoL-AD is a 13-item questionnaire designed to provide a rating of quality of life for older adults with cognitive impairments, both from the perspective of the PwD and also from perspective of the caregiver. It has good reliability and validity and takes ~10 min to complete. A 4-point Likert scale is used to rate each item on indicators such as physical health, mental health, social and financial domains, energy, mood, and memory. The measure yields a single mean score ranging from 13 to 52, with higher scores indicating greater quality of life.

#### Secondary wellbeing outcomes for family caregivers

*Depression* was measured using the 9-item depression scale of the Patient Health Questionnaire (PHQ-9) (47). This is a short-form general health survey designed to screen for depression with good reported reliability and validity (47). Total scores range from 0 to 27 with scores of 0–4 indicating no depression, 5–9 mild depression, 10–14 moderate depression, 15–19 moderately severe depression, and 20–27 indicating severe depression. We also wanted to measure positive aspects of wellbeing for FCGs such as *life satisfaction* and *flourishing*. We used the Satisfaction with Life Scale (SWLS) (48), which has good construct and content validity, test-retest reliability, and internal consistency (49), and the Flourishing Scale (50). The SWLS has 5 items rated on a 7-point scale giving a total score range of 7–35. The Flourishing Scale is an 8-item measure of psychological wellbeing, specifically self-perceived success in areas such as relationships, self-esteem, purpose, and optimism and has good psychometric properties (50). Each item is scored on a 7-point scale, with a total score range of 8–56.

In order to examine *perceptions of the caregiving role* we used the Positive Aspects of Caregiving Questionnaire (PACQ) (51), which consists of nine statements about the caregiver's affective state in relation to the caregiving experience. Each item begins with the stem “Providing help to (name) has…” followed with specific items such as “made me feel useful” and “enabled me to appreciate life more.” Each item is rated on a 5-point ordinal scale ranging from 1 (disagree a lot) through 5 (agree a lot) with a total score range of 9–45.

### Post-intervention interviews

Participant dyads completed semi-structured interviews after attending 20 weekly TGS sessions. Specifically, we asked participants to describe their experience of the group singing sessions and completing the outcome measures. We also asked about any expectations that they had prior to commencing, whether these were met, and whether they had any suggestions for improvement. All interviews were audio recorded and transcribed for analysis.

### Analysis

Recruitment, participation, and completion rates were recorded and presented as percentages. Quantitative data were summarized as mean (standard deviation) and analyzed using repeated measures analysis of variance and paired sample *t*-tests using a significance level of 0.05. Appropriateness of outcome measures was determined by examining response and completion rates and interview responses. Effect size calculations on score changes over time on the outcome measures were also analyzed to ascertain whether the measures were sensitive to change. As a pilot feasibility study with small sample size and no control group, we did not set out to test the effect of the intervention, but rather examine sensitivity and suitability of the outcome measures and the acceptability of the intervention protocol. Qualitative data were analyzed using inductive thematic analysis (52). Intervention acceptability was determined by examining completion rates and interview comments.

## Results

Of the 20 PwD/FCG eligible dyads living in the community who registered interest in the project, 12 dyads consented to participate and enrolled in the study (60% participation rate). It took 4 months to recruit the 12 dyads and their demographic details are presented in Table 1 below. Reasons for non-enrolment included deterioration of the PwD and/or admission into residential care (*n* = 5), or unavailability on the day that the Remini-Sing group intervention was scheduled (*n* = 3). The MMSE scores indicated a range of dementia severity from mild (*n* = 7) to moderate (*n* = 5) and severe (*n* = 1). Mean DRS-2 total scores were well below the normal range of 130–144 for this age cohort (53), as to be expected for PwD.

**Table 1:** Baseline demographics.

	**PwD Mean**	***SD***	**Range**	**FCG Mean**	***SD***	**Range**
Female (male)	7 (5)			6 (6)		
Age—years	77.9	9.9	57-89	73.9	10.1	58–88
MMSE	19.1	4.8	10–26	n/a		
DRS-2 total	102.4	20.7	68–130	n/a		

*MMSE, Mini Mental State Exam; DRS-2, Dementia Rating Scale 2nd edition*.

Nine of the 12 enrolled PwD/FCG dyads completed 20 therapeutic group singing sessions, all assessment time points and post-intervention interview (75% completion rate). Three dyads withdrew before completing 20 sessions due to: death of the PwD participant (*n* = 1), ill health (*n* = 1), or ceased attending after mid assessment (*n* = 1). Eight dyads who completed 20 Remini-Sing sessions were in a spousal relationship and one PWD was cared for by her daughter. Musical history was mixed with three PwD/FCG dyads where both had choral/singing experience, four dyads where one partner (PwD = 3, FCG = 1) had choral/singing experience, and two couples who had never sung together before.

We used a staggered recruitment process where participants commenced and completed their 20 sessions on differing dates over a 12-month period. We chose this recruitment process as it took some time to get sufficient enrollments. Also, we wanted to test the sensitivity of the quantitative measures and to give this the best chance of success we needed to ensure that each participant had attended the same number of sessions. Each participant completed their mid and final assessments after attending their 10 and 20th Remini-Sing session. The nine dyads who completed the final assessment took different lengths of time to complete 20 sessions: 20 weeks (*n* = 1), 21 weeks (*n* = 1), 22 weeks (*n* = 2), 23 weeks (*n* = 1), 24 weeks (*n* = 1), 26 weeks (*n* = 3; 2 dyads had a 6-weeks holiday during the intervention period and one PwD deteriorated and required hospitalization for 5 weeks).

### Quantitative results

Results of the repeated measures analysis of variance and paired sample *t*-tests analyses for the quantitative measures revealed no significant differences between time points for any of the measures used. Most outcomes revealed positive scores at baseline that were sustained over the 20-week intervention period. Table 2 presents the self-rating scales completed by the PwD. There were small effects sizes suggesting that PwD perceived increased relationship quality (baseline-mid d = 0.24, baseline-post d = 0.27), and decreased quality of life (baseline-mid d = −0.35, post point d = −0.20).

**Table 2:** PwD self-rating results (*n* = 9) presented as mean (standard deviation).

**Outcome**	**BL**	**Mid**	**Post**	**Mid-BL**	**Post-BL**	**Post-Mid**
QCPR	61.6 (8.6)	63.2 (7.1)	63.7 (7.4)	1.7* (−4.9 to 8.2)	2.1* (−4.8 to 9.0)	0.4* (−1.4 to 2.3)
AES	27.7 (5.3)	30 (13.2)	28 (8.4)	2.3 (−11.3 to 15.9)	0.3* (−7.3 to 8.0)	−2.0 (−16.1 to 12.1)
QoL-AD	41.4 (2.3)	40 (4.2)	40 (5.3)	−1.4* (−7.4 to 4.6)	−1.3 (−6.6 to 4.1)	0.1* (−7.3 to 7.6)

*BL, Baseline; QCPR, Quality of Caregiver Patient Relationship; AES, Apathy Evaluation Scale; QoL-AD, Quality of Life—Alzheimer's Disease*.

* *Small effect size*.

Table 3 presents the results of assessments completed by the FCG about their PwD. There were no significant differences between time points. There were small effects sizes suggesting that FCG perceived decreases in PwD anxiety (baseline-mid d = −0.38, baseline-post d = −0.28), and a medium effect size for mid-point increase in apathy (d = 0.45). Quality of life was perceived as lower by FCGs than reported by the PwD and also decreased slightly over time. The mean anxiety scores for each behavior measured on the CMAI-SF at each time point ranged from 1 (never) to 2 (less than once a week), with the exception of item 10 (repetitive sentences, calls, questions or words), which moved from a mean of 2.8 to 2.4 (a rating of 3 = once or several times a week).

**Table 3:** FCG rating of PwD results (*n* = 9) presented as mean (standard deviation).

**Outcome**	**BL**	**Mid**	**Post**	**Mid-BL**	**Post-BL**	**Post-Mid**
RAID	12.6 (13.3)	8.3 (4.8)	7.2 (5.5)	−4.2* (−14.8 to 6.4)	−5.3* (−18.9 to 8.2)	−1.1 (−7.7 to 5.5)
AES	37.1 (11.0)	40.3 (14.0)	41.2 (10.6)	3.2^#^ (−5.3 to 11.8)	4.1 (−1.5 to 9.7)	0.9 (−5.2 to 7.0)
QoL–AD	37.2 (5.5)	36.6 (4.5)	35.4 (4.9)	−0.7 (−3.7 to 2.3)*	−1.8 (−7.7 to 4.1)	−1.1 (−4.5 to 2.2)

*RAID, Rating Anxiety In Dementia; AES, Apathy Evaluation Scale; QoL-AD, Quality of Life—Alzheimer's Disease*.

* *Small effect size*,

# *medium effect size*.

On the three outcomes that were completed by both FCG and PwD (QCPR, AES, and QoL-AD), we conducted independent samples *t*-tests to determine differences between scorers. The AES comparisons revealed a significant difference between PwD and FCG scores at baseline (9.4, *p* = 0.039) and post intervention (13.2, *p* = 0.010). The QoL-AD comparisons revealed significantly different scores between PwD and FCG at post intervention (5.2, *p* = 0.043). There were no significant differences between PwD and FCG scores on the QCPR.

Table 4 presents the FCG self-report measure results. Again, there were no significant differences between time points.

**Table 4:** FCG self-rating results (*n* = 9) presented as mean (standard deviation).

**Outcome**	**BL**	**Mid**	**Post**	**Mid-BL**	**Post-BL**	**Post-Mid**
QCPR	57.3 (9.8)	59.7 (7.8)	56.2 (8.6)	2.2^#^ (−2.4 to 6.9)	−1.1 (−8.7 to 6.4)	−3.3 (−7.9 to 1.2)
SWLS	23.8 (6.8)	26.9 (6.6)	28.3 (6.7)	3.1^#^ (−5.5 to 11.7)	4.6* (−3.1 to 12.2)	1.4 (−2.6 to 5.5)
PACQ	31.7 (9.5)	30.8 (5.5)	26.4 (10.2)	−0.9 (−6.5 to 4.7)	−5.2^#^ (−14.3 to 3.9)	−4.3^#^ (−12.3 to 3.6)
PHQ−9	4.7 (5.2)	5.8 (5.5)	4.7 (4.0)	1.1 (−1.7 to 3.9)	0.0 (−3.1 to 3.1)	−1.1 (−4.6 to 2.4)
FS	46.2 (6.7)	46.6 (6.8)	47.2 (6.9)	0.3^#^ (−3.2 to 3.9)	1.0 (−2.3 to 4.3)	0.7 (−1.8 to 3.2)

*QCPR, Quality of Caregiver Patient Relationship; SWLS, Satisfaction With Life Scale; PACQ, Positive Aspects of Caregiving Questionnaire; PHQ-9, Patient Health Questionnaire−9; FS, Flourishing Scale*.

* *Small effect size*,

# *medium effect size*.

Lower scores for relationship quality were reported by FCGs than PwD, with a medium effect size for mid-point increase (d = 0.65) but this decreased again at post intervention. Satisfaction with life scores increased over time (medium effect at mid-point, d = 0.51, and small effect at post-point, d = 0.41), but the positive aspects of caregiving scores were lower post intervention (medium effect, d = −0.56) Depression scores were low at baseline and remained relatively stable. Flourishing scores were high at baseline and also remained relatively stable.

### Qualitative results

Results of the full thematic analysis of the qualitative interview data is published elsewhere (36). For the feasibility investigation, which is the purpose of the current paper, we will explore the qualitative results that pertain to the participants' experience of the Remini-Sing therapeutic singing group intervention, of participating in research and their experience of completing the quantitative measures. It was clear from the participant interview data, that they enjoyed the group sessions and were able to highlight specific aspects of the intervention that were positive. They appreciated the opportunity to sing both familiar songs and learn new songs, harmony parts, and rounds that were perceived as cognitively stimulating.

*We sing the sort of songs that I like to sing (PwD1). We're learning lots of things… Singing in different ways aren't we. We're learning to use the instruments and that's something new isn't it (FCG12)*.

There were several practical elements of the intervention delivery that participants highlighted as supportive, such as the use of power point display for lyrics, the size of the group, and the opportunity for social connection over afternoon tea following the sessions.

*I'm glad they've got the things up on the boards because… I get my words wrong a lot of the time (PwD8). It's sort of the size of the group as well. Um, you can get around everybody. Whereas if it was any bigger, I think that might cause some problems. We perhaps wouldn't be as close to them as we are with these people (FCG10). Getting together afterwards… everyone does appreciate that catching up on how everyone's week's been. And the longer the group is together, then the more that [social connection] really works I think. That's really important because we form special bonds (FCG12)*.

The acceptability of the intervention was most clearly indicated by participants' concerns about the group finishing at the end of the funded research period.

*Everybody's talking about when's it going to finish. And they're not just talking about it—they're really concerned about it. I know it's research—and I know it's incredibly important and I think it's wonderful that its happening, but I think it's such a shame that when the people are in the here and now, that they're actually benefitting from it. It's like being given a trial drug and then it fixes you but you can't keep going (FCG2). I would like that the group goes for ever and ever. That would be very nice (FCG9)*.

In terms of completing the quantitative measures, mostly participants were quite happy to complete these, as they knew it would help the research. The quantity of measures was tolerated well.

*I accept that there was a lot. But I think that if we're doing this for research then it's important, then I don't mind doing it…Besides we had you asking us the questions. It was fun. Remember our first session, you rang me and you said it would probably take half an hour, but it took much longer…I think that might have been us because we were having so much fun (FCG2). Oh, they were a bit hard. Because sometimes people can explain themselves and some can't… [PwD10] doesn't remember. He doesn't remember too much about things like that… [but] we felt that we've got to give it go and that it is answering all the questions so you've got everybody's point of view (FCG10)*.

Some of the measures had positively and negatively worded questions (particularly the QCPR) and this was mentioned as being difficult by several participants.

*Oh look. There were a lot of questions. You just have to be on alert because some of them are looking at it from one way and others from the other way that you're not. So you have to concentrate. But yes, they're fine (FCG12). The chopping and changing from one side to the other—you know you're sort of in one frame—but then you actually have to read the question again to make sure you've answered it correctly. I was about to put the wrong answer and had to think again (FCG4)*.

One participant particularly disliked the PACQ as she didn't equate her self-worth with her role as a caregiver for her husband.

*The one about the positive aspects of caregiving. I thought—look – I could have torn that up because I thought my worth as a person is not tied up to being a caregiver so I did struggle with answering that cause I just thought I would like to put a line right through—but I thought, well I know I need to answer this… but then you might really want that as part of your research, I'm not sure (FCG12)*.

Several of the measures were difficult to complete for the PwD with more advanced dementia.

*I can tell that most of them, he doesn't understand what it is. Mostly he goes backwards (FCG9). [PwD4] can't remember those 5 [answer options] – the nuances are too much. Too fine for a person with dementia. But good that we see both sides of the relationship. Each one of these forms comes from a different source—I realize that—but each one uses different terminology, which is hard (FCG4)*.

A participant with semantic dementia was concerned she would have difficulty with completing the measures but felt supported by the researchers to do this successfully.

*When we came first to you—and um—I was ah—when they said they had to um…you do all these writings—um—I said to [FCG8]—oh we'll just be put out and I'll be very disappointed. And anyway, you led me to do it properly and ah—ever since then—it's our day! (PwD8)*.

## Discussion

As a pilot feasibility study, aim was not to measure efficacy of the intervention, but rather to determine the acceptability of the intervention and the research protocol. We also wanted to determine whether the quantitative measures were appropriate, sensitive to change, and acceptable to participants. We aimed to examine feasibility by testing the intervention and the assessment measures with our target population and asking them how they experienced both.

The accessibility and acceptability of this therapeutic group singing intervention for PwD and their FCGs living in the community was good. Attendance rates were relatively high considering the potential hurdles to group attendance imposed by dementia diagnoses and caregiver roles. Aside from the two couples who went on a 6-week holiday during the intervention period, most dyads only missed between 0 and 4 weeks during the 20-week intervention. Two of the dyads who withdrew, did so due to ill health or death, and one dyad who completed the study missed several sessions due to dementia progression and subsequent hospitalization. Health status was therefore a major factor that impacted attendance in this study.

The primary outcome of *relationship quality* (as measured by QCPR scores) was scored relatively highly at baseline and did not decrease over the 20-week intervention period. The QCPR scores reported by FCGs were similar to those reported in other dementia-focused studies (40). These sustained high scores for relationship quality are noteworthy in the context of the significant burden placed by a diagnosis of dementia on both members of the relationship dyad. It is interesting to observe that the PwD rated relationship quality higher than FCGs at all time points. This could indicate cognitive decline and decreased insight related to dementia and thus reduced ability to accurately judge relationship quality and perhaps overestimate relationship quality. The difference in PwD and FCG scores on the QCPR could also reflect the increased burden of caregiving placed on the relationship as perceived by the FCG.

*Anxiety* of the PwD was rated by their FCG using the RAID, where a score of 11 or more indicates significant clinical anxiety (42). The reduction in PWD anxiety from a mean baseline RAID score of 12.6 to 8.3 at mid-assessment and 7.2 at post-assessment, therefore represents an important clinical difference, although the sample size/power was too low to achieve statistical significance. Mean *apathy* scores were low at baseline and remained low over the course of the intervention. Again, it was interesting to see that FCGs rated apathy significantly higher than the PwD themselves, and the mean post-intervention caregiver-rated AES score was approaching the threshold of 42, indicating mild apathy. *Agitation* behaviors (as observed by FCGs using the CMAI-SF) were very low to non-existent at baseline and remained low over the course of the intervention period. Self-reported *quality of life* (as measured by the QoL-AD) was high at baseline and remained stable over the 20-weeks of the study, however FCGs rated their loved one's quality of life as much lower. Logsdon et al. (54) also reported a similar phenomenon where FCGs consistently rated PwD quality of life lower than the PwD themselves. They suggested that this discrepancy (which has also been reported in other clinical populations) could be because caregiver QoL-AD ratings may be lower due to bias caused by their own levels of burden or depression (54). Caregiver burden was not measured in our study, but low depression scores and high SWLS scores suggest that Logsdon's suggestion may not explain the differences we saw in QoL-AD scores. Our study results support previous research indicating that people with mild to moderate dementia can rate their own quality of life and that caregiver ratings do not substitute for self-ratings of quality of life (54). Previous research suggests that behavioral and psychological symptoms of dementia can be present even in the early stages of dementia (55, 56). However, given the appearance of floor and ceiling effects on many of the measures utilized in this study, it may be that the participants who volunteered for this therapeutic group singing study were not actually struggling with these symptoms yet. We cannot generalize from such a small sample though, so a larger randomized, controlled trial is needed to determine characteristics and needs of community-dwelling PwD/FCG dyads who volunteer to participate in music therapy research.

There was likely a floor effect for FCG *depression* (as measured by the PHQ-9) as this was very low at baseline and remained low across the intervention period. *Satisfaction with life* improved slightly and *flourishing* scale scores remained high from baseline. It is possible that FCG participants did not relate life satisfaction and flourishing to their caregiver roles. The *positive aspects of caregiving* scale scores did not change much from pre to post-intervention. As stated by one participant, it may be that the FCGs did not equate their self-worth with their role as a caregiver. Although we had intentionally attempted to look at the positive aspects of caregiving, rather than caregiver burden, based on the participant experience of the PACQ, we would choose to examine caregiver burden in a future study. Further, one of the major themes that emerged from our full qualitative analysis (36) was the effect of the group singing intervention on building new supportive relationships. Therefore, we would also include a measure of social connectedness in future studies in this area.

Given the overall positive wellbeing scores at baseline, it is possible that this study attracted participants who were already coping well with their dementia diagnosis or caregiving role. Similarly, the relationship quality scores were high at baseline and remained high, thus creating a potential ceiling effect. One of the FCGs commented on this in relation to completing the QCPR measure.

*I think if you've got a solid relationship before all this starts then it's probably not going to vary a great deal—but then I might be wrong in that. That's how it is for us, but it could be very different for others. Because it's certainly—it can be a testing time (FCG12)*.

There was an alteration to the intervention protocol, as during the course of the study we realized that our planned home music program was not being utilized by participants. This may have been due to the fact that the PwD who were enrolled in our study were not yet displaying behavioral and psychological symptoms of dementia that our home program playlists were designed to address. Further, it became apparent that the home music program and education about how to best use recorded music in dementia care, was actually a separate intervention in and of itself and was more likely to be of use in dealing with more advanced stages of dementia. Instead, we began to give participants recordings of songs that we were singing in the group sessions. This enabled and encouraged participants to continue to sing together outside of the group sessions and helped them to learn new songs and harmony parts. Participants reported that these recordings were very useful and did indeed encourage them to sing together outside of the group sessions and helped them to practice new musical material.

## Limitations

This feasibility study used a single group pre-post design. As such, it was never intended to measure efficacy of the therapeutic group singing intervention, but rather to examine acceptability of the intervention and assessments. As we did not have a control group, we were not able to determine retention rates for participants allocated to a usual care or wait-listed control condition. The small sample size and heterogeneity of participants in terms of age, dementia severity and caregiver relationship also mean that results should be interpreted with caution.

Authors 1 and 2 who delivered the group intervention also conducted the assessments and interviews. As a non-controlled study, blinding was not really relevant however, and we considered it an important part of the feasibility testing for the primary researchers to gain an understanding of how participants managed the quantitative assessments. Further, we intentionally drew upon this pre-existing therapeutic relationship and the subsequent trust and familiarity participants shared with the researchers to elicit rich data in the post-intervention interviews. It is possible that participants could have been less likely to reveal negative experiences in their interviews as a result of this dual therapist/interviewer role. However, we did receive several participant examples of negative experiences and frank reflections on the research participation experience suggesting that the trust developed instilled freedom of expression in participants.

The social aspects of group singing attract people who are wishing to connect with others but may be aversive to those who are depressed or socially withdrawn (common experiences for both PWD and FCG). This phenomenon might explain our ceiling effects. It is possible that the focus on singing may exclude people who don't like music or singing, or feel that they are not musically talented. However, our qualitative results suggested that the focus on singing was what drew people to the study. Specifically, some participants reported feeling lack of confidence about their singing ability although they were all willing to give it a go. Our group singing intervention may have also provided them with an opportunity to form social connections with other people living with dementia, with a focus on music rather than dementia. Moreover, the accessibility of singing throughout the dementia trajectory, together with the social, emotional and cognitive benefits make it an ideal focus for group intervention in dementia.

## Conclusion

The Remini-Sing therapeutic group singing intervention and research protocol were acceptable to the participants in this study and feasibility was thus demonstrated. As discussed, there were a few minor things that we would change in future studies, such as the focus of the home music program and some changes to quantitative measures selected. In particular, for the FCGs we would replace the SLWS and Flourishing scale with a measure of health-related quality of life and exchange the PACQ with a traditional measure of caregiver burden. For the PwD, the measures of apathy and agitation were not sensitive to change in this very small community-dwelling cohort, so we would replace these with a dementia-specific measure of depression. The medium effect size observed for reductions in anxiety indicate that the RAID may a useful measure to retain. Similarly, relationship quality and social connectedness would be outcomes to measure in future fully powered studies, as supported by the qualitative data in this feasibility trial.

According to participant feedback, the success of the intervention was multifaceted. The combination of singing familiar, favorite songs, with the cognitive challenge of learning new musical material, in a supportive and nonjudgmental environment, made the groups accessible, enjoyable and therapeutic. Clearly such community-based, dyad-focused therapy interventions hold great potential to fill an important need for social connection and support, as well as addressing personal wellbeing and quality of life for community-dwelling PwD and their FCGs.

## Author contributions

JT was responsible for the overarching design and conduct of the study. JT and IC designed and delivered the intervention, conducted quantitative assessments, qualitative interviews, and analysis of all data. FB assisted with the qualitative analysis and interpretation of quantitative data. Y-EL assisted with selection of appropriate outcome measures and advised on administering the measures and interpretation of results. All authors contributed to the writing of the manuscript.

### Conflict of interest statement

The authors declare that the research was conducted in the absence of any commercial or financial relationships that could be construed as a potential conflict of interest.
